# Subjective Memory Complaints in Portuguese Young Adults: Contributions from the Adaptation of the Prospective and Retrospective Memory Questionnaire

**DOI:** 10.5334/pb.387

**Published:** 2018-04-27

**Authors:** Diana R. Pereira, Pedro B. Albuquerque

**Affiliations:** 1 Neuropsychophysiology Lab, CIPsi, School of Psychology, University of Minho, Braga, PT; 2 Human Cognition Lab, CIPsi, School of Psychology, University of Minho, Braga, PT

**Keywords:** assessment, memory complaints, PRMQ, prospective memory, psychometric study

## Abstract

Self-report instruments that allow to characterize the frequency of daily memory failures are essential for a comprehensive assessment of memory functioning. In this context, we aimed to provide preliminary evidence of validity and reliability for the European Portuguese adaptation of the Prospective and Retrospective Memory Questionnaire (PRMQ). A total of 1052 healthy participants completed an online survey with the PRMQ. The exploration of the construct validity suggested the tripartite model with a general memory, a prospective memory, and a retrospective memory factors to have the best adjustment to the data. Measurement invariance across age and sex groups was also verified. The questionnaire revealed good convergent validity with a general self-report measure of memory (0.778 < *r* < 0.853), and satisfactory values of internal consistency (0.779 < Cronbach’s alpha < 0.887) and of test-retest reliability (0.815 < *r* < 0.852). There were no prominent effects of sex and age in the PRMQ scores. Although the sample encompassed mainly younger and highly educated adults, this study presented the first evidence of validity and reliability for the European Portuguese version of the questionnaire.

## Introduction

Memory lapses are common events in our daily lives, and subjective memory complaints are present across lifespan, even if with distinct qualitative features and with higher prevalence among the elderly (Bassett & Folstein, 1993; Dobbs & Rule, 1987; Ginó et al., 2010; Mendes et al., 2008; Pearman, 2009; Ponds, Commissaris & Jolles, 1997). In the case of older people, subjective memory complaints have been particularly associated with the management of daily living activities and perceived quality of life, and they have been studied as possible indicators of cognitive decline, mild cognitive impairment (MCI) and even dementia (e.g., Farias et al., 2009; Hong et al., 2014; Juncos-Rabadan et al., 2012; Maki et al., 2014; Montejo et al., 2012; Ogata et al., 2015; Rönnlund et al., 2015a; Rönnlund et al., 2015b; Waldorff et al., 2012). Despite the great importance of subjective memory complaints in elderly populations, the characterization of the same phenomenon in younger groups is also relevant to explore how memory changes are perceived across development, and how this self-report can be used in the evaluation of memory difficulties in specific age groups (Ginó et al., 2010; Pearman, 2009).

More than forgetting information about past events, research suggests individuals tend to report forgetfulness for previously planned intentions when the appropriate circumstances arise, that is they perceive prospective memory (PM) failures as more frequent than retrospective memory (RM) failures (Crawford et al., 2003; Hsu & Hua, 2011; Smith et al., 2000; Terry, 1988). Nevertheless, the number of reports dedicated to the development and study of psychometric properties of PM measures is inferior compared to RM measures (Thöne-Otto & Walther, 2008; Woods et al., 2008). This scenario has been changing in recent years due to the development of different objective and subjective measures that integrate both PM and RM functioning. One chief example is the Prospective and Retrospective Memory Questionnaire (PRMQ; Smith et al., 2000), in which the individuals are invited to subjectively judge the frequency of specific RM and PM lapses.

This brief questionnaire has been extensively applied in different cultural backgrounds, and in distinct healthy and clinical populations, revealing good psychometric properties (see Supporting Table S1). Even so, the study of how specific sociodemographic variables, namely sex and age, are related with the scores obtained in the PRMQ has been variable across studies and different cultural adaptations. While some studies revealed no differences between male and female participants (Crawford et al., 2006; Hsu & Hua, 2011; Rönnlund, Mäntylä & Nilsson, 2008; van der Werf & Vos, 2011), others found that male participants reported more RM (Crawford et al., 2003) or PM slips than female participants (Crawford et al., 2006). Additionally, in the study of Piauilino and colleagues (2010), the female participants showed more general memory failures than male participants. Concerning age, it is common to find no significant associations between age and PRMQ scores (Crawford et al., 2006; Crawford et al., 2003; Piauilino et al., 2010; Smith et al., 2000; van der Werf & Vos, 2011) or weak correlations between some scores (González-Ramírez & Mendoza-González, 2011; Hsu & Hua, 2011). Some of the former findings can be a result of large sample sizes (e.g., Crawford et al., 2003; González-Ramírez & Mendoza-González, 2011), since p-values are amenable to sample size variability (Cohen, 1990; Sullivan & Feinn, 2012). Hence, the general picture indicates that sex and age might not have a prominent influence in the PRMQ.

The main aim of this work is to provide, for the first time, data regarding the adaptation and the psychometric properties of the European Portuguese version of the PRMQ with a web-based sample of younger healthy participants. We also intend to probe how sex and age might play a role in the results obtained in this version. For this purpose, an analysis of measurement invariance is included to test if the concepts of the PRMQ are being measured in a similar way in distinct sex and age groups of the current sample.

## Methods

### Participants

A total of 1052 healthy participants, aged between 18 and 54 years old (*M* = 22.24, *SD* = 5.59), 71.6% female, and with an average of 14.24 years of formal education (*SD* = 2.37) collaborated in this study. A subset sample (*n* = 236) completed again the assessment with an average interval of 13.87 days (*SD* = 3.46). They were aged between 18 and 52 years (*M* = 23.28, *SD* = 5.72), 74.2% female, and had an average of 14.88 years of formal education (*SD* = 2.56).

This study was approved by the Ethics Subcommittee for Social and Human Sciences (SECSH) of University of Minho, and all the participants provided informed consent prior to their enrollment.

### Instruments

The assessment included a brief sociodemographic questionnaire and two memory questionnaires, the PRMQ (Smith et al., 2000) and the Questionnaire of Memory Lapses (QML; Pinto, 1990). The PRMQ is a short questionnaire composed of 16 items in which the participants are invited to evaluate the frequency of occurrence of distinct memory failures using a 5-point rating scale (1 – never; 5 – very often). It comprises two subscales with eight items each, a PM subscale (e.g., “Do you decide to do something in a few minutes’ time and then forget to do it?”) and a RM subscale (e.g., “Do you fail to recognize a place you have visited before?”). The total score corresponds to the sum of all items, which ranges between 16 and 80. Higher scores depict higher frequency of perceived and reported memory slips. The values of internal consistency (between 0.71 and 0.97) and of test-retest reliability (between 0.78 and 0.81) are adequate and above 0.70 as recommended (Burlingame et al., 1995) for different cultural adaptations (see Supporting Table S1).

The QML (Pinto, 1990) is a Portuguese measure with 36 items that evaluates the frequency of daily memory slips in a 7-point scale (1 – never; 7 – always). The score is based on the mean response of all items. The questionnaire has a good internal consistency, namely a good Cronbach’s alpha (total scale = 0.96) and a good split-half reliability (total scale = 0.92) (Vaz, Daniel & Vicente-Castro, 2011). The test-retest reliability was between 0.35 (item 19) and 0.90 (item 34), the average correlation amid items was 0.61 and regarded as acceptable (Pinto, 1990).

### Procedure

The translation of the PRMQ was based on the proposal of Cha, Kim and Erlen (2007) by using the combination of the translation/back-translation method, the committee approach, and the pretest procedure. After having the authorization from the responsible author, the translation from English to European Portuguese was independently conducted by three translators. The translated versions were then discussed in group to reach a single version by agreement of all members. The structure of most items was preserved, yet item 10 – “Do you intend to take something with you, before leaving a room or going out, but minutes later leave it behind, even though it’s there in front of you” – was an exception, because it suffered a significant change in the order of the words. This unique version was tested and discussed in a small group of participants (*n* = 10) to check the clearness of linguistic features and content. Small linguistic changes were incorporated in this phase. The back-translation was then performed by a Portuguese researcher that lived in the United Kingdom, and the resultant translation was compared with the original version to assure content equivalence. No major issues were observed in this stage. Finally, a pilot study was conducted with 16 university members to test, once again, the comprehensibility and the cultural equivalence of the items of the questionnaire.

Regarding the web-based procedure, the PRMQ and the QML were firstly converted to an online survey using the Google Forms (Google Inc., California, USA, 2016). The survey included four sequential parts. First the informed consent wherein only the participants that gave consent had access to the subsequent parts, second the sociodemographic and the health-related questions, third the PRMQ, and fourth the QML. In the last part of the QML, we also invited the participants to complete again both questionnaires with a temporal interval of approximately 10 days. The dissemination of this study was mainly achieved using social networking websites and with the aid of some Portuguese universities.

### Data analysis

The construct validity was tested with a confirmatory factor analysis (CFA), and the selected models were based in previous studies (Crawford et al., 2006; Crawford et al., 2003; González-Ramírez & Mendoza-González, 2011; Hsu & Hua, 2011; Piaulino et al., 2010; Rönnlund, Mäntylä & Nilsson, 2008). Three models were scrutinized: Model 1 that considered a general episodic memory factor underpinning covariance among the 16 items; Model 2 that deemed the existence of two associated factors, a PM and a RM factor; Model 3 that proposed an orthogonal tripartite approach with a general episodic memory, a PM factor which included all the PM items, and a RM factor comprising the RM items. The maximum likelihood (ML) method was used to estimate all the parameters, since there is evidence showing that it can be robust to moderate violations of normality (Hu, Bentler & Kano, 1992; Muthén & Kaplan, 1985), offering also acceptable results for ordinal variables (Rhemtulla, Brosseau-Liard & Savalei, 2012). Different fit indices were used as indicators of good fit, namely a root mean squared error of approximation (RMSEA) value inferior to 0.06, a standardized root mean squared residual (SRMR) value below to 0.08, and a comparative fit index (CFI) above or equal to 0.95 (Bentler & Moijart, 1989; Hu & Bentler, 1999).

For the model with the best fit indices, we also performed a multi-group CFA (MGCFA) with the ML method to examine the measurement invariance (MI) across sex and age. The female group was composed of 753 participants and the male group had 294. The age groups were formed by diving the sample in three approximately equal parts (*n* = 372/375/304), using 19 and 22 years as cutoffs. The idea was to verify if the constructs were being measured in a similar way in the former groups by applying a series of gradually stringent invariance tests (van de Schoot, Lutlig & Hox, 2012): configural invariance, metric invariance, scalar invariance, uniqueness MI, and structural invariance. To evaluate the goodness of fit across MI models, a change of ≤0.01 in CFI (ΔCFI) and of ≤0.015 in RMSEA (ΔRMSEA) were used as recommended invariance indicators (Chen, 2007; Cheung & Rensvold, 2002). The convergent validity was tested with a Pearson’s product-moment correlation between the PRMQ scores and the mean responses in the QML.

Regarding the reliability features of the PRMQ, the internal consistency was explored by using the Cronbach’s alpha, and the test-retest reliability was based on Pearson’s correlations between the first and the second moment of assessment in a subgroup of participants. The influence of sociodemographic variables was tested by applying Pearson’s correlations for age and independent samples t-tests for sex. The interpretation of all correlation coefficients followed the literature standards (Cohen, 1988; Mukaka, 2012). Additionally, we also performed a paired-sample t-test to evaluate if there was any statistically significant difference between the frequency of reported RM and PM failures.

The results were interpreted as statistically significant for *p* < 0.05, and we reported Cohen’s *d* as a measure of effect size for t-tests (Cohen, 1988; Sullivan & Feinn, 2012). The statistical analyses were conducted with the IBM Statistical Package for the Social Sciences Statistics software, version 22 (IBM SPSS Statistics for Windows, Armonk, NY), and with the AMOS software, version 23 (IBM SPSS AMOS, Chicago).

## Results

The first step in the data analysis was to obtain the descriptive statistics for each item of the PRMQ, and to assure that all the values of the 5-point rating scale were used in each item (see Table 1).

**Table 1:** Descriptive Statistics of the Items of the Prospective and Retrospective Memory Questionnaire and Values of the Standardized Loads for the Tripartite Model.

Items	*M*	*SD*	Skewness	Kurtosis	General episodic memory factor	PM factor	RM factor

1	3.03	0.86	0.40	–0.10	0.62	0.98	
2	1.96	0.81	1.06	1.97	0.33		0.28
3	2.53	1.00	0.42	–0.22	0.65	0.19	
4	2.92	0.96	0.30	–0.27	0.62		0.21
5	2.70	1.04	0.36	–0.49	0.55	0.04	
6	1.62	0.78	1.35	2.00	0.28		0.29
7	2.13	0.91	0.64	0.17	0.60	0.05	
8	2.36	0.97	0.60	0.17	0.55		0.42
9	2.48	1.00	0.58	0.08	0.50		0.14
10	2.60	0.99	0.48	–0.09	0.66	0.01	
11	2.45	1.04	0.62	–0.09	0.64		–0.06
12	2.63	0.94	0.48	0.11	0.63	–0.01	
13	2.15	0.93	0.64	0.10	0.55		0.24
14	2.47	1.02	0.47	–0.30	0.61	–0.01	
15	2.21	0.98	0.69	0.22	0.48		0.45
16	2.88	0.94	0.43	–0.03	0.64	0.13	

### PRMQ validity

#### Construct validity and measurement invariance

A summary of the fit indices of the three models can be found in Table 2. For all the models, we observed that the chi-square (χ^2^) statistic was statistically significant (*p* < 0.05), which suggests a poor fit to the observed data. Even so, the χ^2^ is recognized to be highly sensitive to sample size, hence it is difficult to retain the null hypothesis. In this case, the analysis of the fit indexes is essential. More specifically, the Model 1 showed a poor fit with a CFI below 0.95 and a RMSEA above 0.06. The standardized item loadings varied between 0.33 and 0.67, and the loading average was 0.57 (see Supporting Figure S1). A similar result was found for Model 2, the CFI was inferior to 0.95, and the RMSEA marginally superior to 0.06. The standardized item loadings ranged between 0.56 and 0.69 for the PM factor (average was 0.63), between 0.36 and 0.67 for the RM factor (average was 0.55), and there was an association of 0.90 between factors (see Supporting Figure S2). The Model 3 that comprised three orthogonal factors, the general episodic memory factor, the RM factor and the PM factor was the model with the best fit indices, namely it yielded a RMSEA below 0.06, a SRMR below 0.08 and a CFI marginally below the cutoff value of 0.95. Despite this, not all the item loadings were satisfactory (see Table 1 and Supporting Figure S3). Indeed, most of the items presented higher loadings in the general episodic factor than in the PM and the RM factors.

**Table 2:** Summary of the Fit Indices for the Models Tested with the Confirmatory Factor Analysis.

Model	χ^2^	*df*	RMSEA	SRMR	CFI	ΔRMSEA	ΔCFI

1. General episodic memory factor	652.956	104	0.071	0.046	0.895		
2. RM and PM associated factors	578.801	103	0.066	0.043	0.909		
3. Tripartite model	352.027	88	0.053	0.033	0.949		
Multigroup analysis across sex						
Configural invariance	457.530	176	0.039	0.035	0.945		
Metric invariance	475.924	205	0.036	0.035	0.947	–0.003	0.002
Scalar invariance	509.614	220	0.035	0.035	0.943	–0.001	–0.004
Uniqueness MI	532.025	236	0.035	0.036	0.942	0	–0.001
Structural invariance	540.648	239	0.035	0.036	0.941	0	–0.001
Multigroup analysis across age						
Configural invariance	557.773	264	0.033	0.040	0.943		
Metric invariance	623.453	322	0.030	0.046	0.942	–0.003	–0.001
Scalar invariance	699.913	352	0.031	0.045	0.933	0.001	–0.009
Uniqueness MI	753.016	384	0.030	0.047	0.929	–0.001	–0.004
Structural invariance	766.583	390	0.030	0.053	0.927	0	–0.002

*Note.* CFI = Comparative Fit Index; df = Degrees of Freedom; MI = Measurement Invariance; RMSEA = Root Mean Squared Error of Approximation; SRMR = Standardized Root Mean Squared Residual.

The results of the MGCFA across sex and age can be accessed in Table 2. Although the χ^2^ statistics were once again significant for all models (*p* < 0.05), the fit indices of SRMR and RMSEA were satisfactory, and the CFI ranged between 0.93 and 0.95, revealing values slightly under the cutoff point. Moreover, the goodness of fit of sex and age MI models was sponsored by a ΔCFI and ΔRMSEA below 0.01. These results support the claim that the PRMQ is an analogous measure in these groups.

#### Convergent validity

The correlations between the average frequency of daily memory lapses obtained from the QML and the PRMQ scores were all satisfactory, positive, and statistically significant (*p* < 0.001). More particularly, the correlation was higher for the total PRMQ (*r* = 0.85), then for the PM subscale (*r* = 0.80), and finally for the RM subscale (*r* = 0.78).

### PRMQ reliability

#### Internal consistency

In Table 3, it is presented the descriptive statistics of the total and the subscales scores of the PRMQ, and the correlations subscale-total and subscale-subscale which were satisfactory. The internal consistency values, estimated with the Cronbach’s alpha, were also acceptable (Burlingame et al., 1995): 0.89 for the total scale, 95% CI [0.88, 0.90], 0.84 for the PM subscale, 95% CI [0.83, 0.85], 0.78 for the RM subscale, 95% CI [0.76, 0.80]).

**Table 3:** Summary of the Descriptive Statistics of the PRMQ Scores and of the Correlations Between Scores.

	*M (SD)*	Range	Skewness	Kurtosis	PRMQ total score	PM score	RM score

PRMQ total score	39.10 (9.26)	17–74	0.54	0.44	–	0.94*	0.92*
PM score	20.98 (5.28)	9–40	0.49	0.21	0.94*	–	0.72*
RM score	18.13 (4.70)	8–37	0.60	0.63	0.92*	0.72*	–

*Note.* PM = Prospective Memory; PRMQ = Prospective and Retrospective Memory Questionnaire; RM = Retrospective Memory; * *p* < .001.

#### Test-retest reliability

From the subset sample that completed the PRMQ in two different assessments, it was possible to obtain positive, strong and statistically significant correlations (*p* < 0.001) between the total PRMQ scores (*r* = 0.85), the PM scores (*r* = 0.83) and the RM scores (*r* = 0.82). These results suggested that the PRMQ has a good temporal stability.

### Effects of sociodemographic variables (sex and age)

Concerning the variable sex, it was possible to observe no statistically significant differences between male and female participants in the total score, *t*(1045) = 1.56, *p* = 0.118, *d* = 0.11, 95% CI [–0.25, 2.23], and in the RM score, *t*(1045) = 0.65, *p* = 0.514, *d* = 0.04, 95% CI [–0.42, 0.84]. Yet there was a significant difference for the PM score, *t*(1045) = 2.16, *p* = 0.031, *d* = 0.15, 95% CI [0.07, 1.49]) wherein female participants reported higher frequency of daily PM related lapses (*M* = 21.18, *SD* = 5.24) in comparison with male individuals (*M* = 20.40, *SD* = 5.28). Even so, the magnitude of the difference was small (Cohen, 1988; Sullivan & Feinn, 2012).

In the case of age, the results yielded statistically significant negative correlations for the total PRMQ score (*r* = –0.09, *p* = 0.004), for the PM score (*r* = –0.09, *p* = 0.003), and for the RM score (*r* = –0.074, *p* = 0.017). However, all these correlation coefficients can be regarded as negligible (Cohen, 1988; Mukaka, 2012; Sullivan & Feinn, 2012).

We further analyzed if there were differences between the frequency of PM and RM failures. A paired-sample t-test revealed that the PM scores (*M* = 20.98, *SD* = 5.28) were significantly superior to the RM scores (*M* = 18.13, *SD* = 4.70), *t*(1051) = 24.63, *p* < 0.001, *d* = 0.76, 95% CI [2.63, 3.08]), still the magnitude of this difference was once more small.

## Discussion

The main goal of this study was to provide validity and reliability evidence for the European Portuguese version of the PRMQ. In accordance with previous studies (see Supporting Table S1), this version showed satisfactory values of internal consistency, as well as good test-retest reliability, asserting the PRMQ as a reliable instrument.

Regarding the construct validity, although the tripartite model was the one with the best fit indices as already found in former investigations (Crawford et al., 2006; Crawford et al., 2003; González-Ramírez & Mendoza-González, 2011; Hsu & Hua, 2011; Piauilino et al., 2010; Rönnlund, Mäntylä & Nilsson, 2008; van der Werf & Vos, 2011), it was also observable that not all the items loaded in the expected factors. A similar pattern was reported in studies using an exploratory factor analysis (Benites & Gomes, 2007; González-Ramírez & Mendoza-González, 2011). So far, only two studies found satisfactory item loadings for the tripartite model (Crawford et al., 2003; Rönnlund, Mäntylä & Nilsson, 2008). Interestingly, in other studies (Crawford et al., 2006; González-Ramírez & Mendoza-González, 2011), the items loaded higher when considering the model with only a general episodic memory factor, even if the fit indices were not satisfactory. Moreover, the Model 2 which showed a good correlation between PM and RM factors also pointed to shared variance among items, supporting an implicit general memory factor (González-Ramírez & Mendoza-González, 2011). Higher item loadings were obtained in this model in comparison with the Model 3 in which the same factors were considered independent. These results can pose some difficulties to the use of the PM and RM factors, hence when using these subscales, it is important to take into account that they gather specific and shared variance (Crawford et al., 2003). In the case of MI across age and sex with the tripartite model, it was possible to find evidence of measurement equivalence between different sample subgroups. Thus, this is one of the first studies showing that the PRMQ can be used and interpreted in a similar way in distinct sex and age subgroups.

The convergent and divergent validity of the PRMQ has been mildly addressed in the literature, yet the results obtained so far seem contradictory. Some studies find associations between the PRMQ scores and other general self-report measures of memory (e.g., Gondo et al., 2010; van der Werf & Vos, 2011), while others report only a relation for the RM subscale and not for the total score nor the PM score (e.g., Benites & Gomes, 2007). In here, we provided some evidence of convergent validity with the QML (Pinto, 1990) for all scores, which fits well with the notion of common variance underlying PM and RM factors. More importantly, the evidence so far shows that the PRMQ has some convergent validity at least when similar self-report measures are used (van der Werf & Vos, 2011; Vestergren et al., 2011), which is not always the case when using objective memory tasks (Lee et al., 2016; Paquet et al., 2017; Thompson et al., 2015; Uttl & Kibreab, 2011).

Considering that we assured by means of MI that the questionnaire was being interpreted in a similar way regardless of sex, no statistically significant differences between male and female participants were observed for the total PRMQ and the RM scores. Even so, a small difference appeared in the case of the PM score, showing higher frequency of PM slips in female than in male participants. A similar outcome was found for the total PRMQ score in the Brazilian study (Piauilino et al., 2010). Since the differences were small, these results can be understood as an effect of sample size (Crawford et al., 2003). Thus, this study supports the idea that sex does not seem to have a prominent role in the self-report of memory failures (Hsu & Hua, 2011; Ponds, Commissaris & Jolles, 1997; van der Werf & Vos, 2011).

In the case of age and after assuring MI in different age subsets of the sample, statistically significant yet weak correlations between age and PRMQ scores were found, probably due to the large sample size, which is in line with previous investigations (Crawford et al., 2006; Crawford et al., 2003; González-Ramírez & Mendoza-González, 2011; Hsu & Hua, 2011; Piauilino et al., 2010; van der Werf & Vos, 2011). From the literature standpoint, this is an intriguing finding because other studies have been supporting the existence of a decline with age in both PM and RM objective measures (e.g., Deary et al., 2009; Zimmermann & Meier, 2006). Furthermore, some studies also convey the idea that subjective memory complaints are more frequently reported in aged groups (e.g., Dobbs & Rule, 1987; Ponds, Commissaris & Jolles, 1997). In the light of this evidence, firstly, some studies reveal that subjective memory complaints do not increase with age (e.g., Mendes et al., 2008; Rowell et al., 2016; Smith et al., 2000), and the results found here lend evidence to such pattern. Secondly, it is important to consider that the literature has also been showing weak associations between objective and subjective measures of memory functioning (Mendes et al., 2008; Paquet et al., 2017; Thompson et al., 2015; Uttl & Kibreab, 2011), hence the same pattern across age may not be verified for both type of measures. Indeed, other variables such as mood seem to be particularly correlated with self-report measures of memory functioning (Bassett & Folstein, 1993; Pearman, 2009), and this result holds true for younger and middle-age adults (Mendes et al., 2008; Rowell et al., 2016). While objective tests seem to provide a more reliable approximation to memory functioning, subjective measures allow to explore other factors, such as memory self-efficacy, self-awareness and mood (Roche, Fleming & Shum, 2002; Rönnlund et al., 2011). In this sense, self-report measures allow the possibility to explore the beliefs underlying personal memory abilities. Also, when persons are aware of their abilities, they tend to implement distinct strategies to prevent possible failures and to assure their success (McDonal-Miszczak, Gould & Tychynski, 1999; Meeks, Hicks & Marsh, 2007). Thus, the information extracted from both objective and subjective measures is diverse and, more importantly, it can complement each other (Fleming et al., 2009; Mioni, McClintock & Stablum, 2014).

Regarding the comparison between the frequency of PM and RM failures, we were not able to replicate the finding that PM problems are more frequently reported than RM problems (Crawford et al., 2003; Hsu & Hua, 2011; Piauilino et al., 2010; Smith et al., 2000), since the difference found was small in magnitude. This result dovetails with the higher item loadings obtained for the general episodic memory factor in all the models tested, suggesting no clear distinction between the evaluation of RM and PM items.

It is noteworthy that only the Dutch adaptation of the PRMQ had an internet-based sample (van der Werf & Vos, 2011), so the present study is the second to use an internet-based procedure. This can raise some issues including the equivalence between web-based and paper-and-pencil approaches, and the sample bias in favor of younger and highly educated individuals. Regarding the first, this study suggests no major differences between procedures, at least in younger adults, since similar outcomes of reliability and validity were obtained. In fact, our team conducted a pilot study with the paper-and-pencil version of the PRMQ in a sample of 85 participants (age: 17–48 years), and it was possible to find good positive correlations between the QLM (Pinto, 1990) and the PRMQ scores (Total: 0.77; PM: 0.79; RM: 0.68; all *p* < 0.001), and good internal consistency (Total: 0.89; PM: 0.90; RM: 0.78). Even so, future studies are warrant to test the equivalence of internet-based and paper-and-pencil procedures. Concerning the sample bias, this is precisely one central limitation. Therefore, the results obtained here are restricted as we did not have a representative sample of the European Portuguese population. Furthermore, this ends up introducing other shortcomings such as the impossibility to study in a more comprehensive manner the influence of age, and even the role of formal education which can be an important factor to consider (e.g., Hsu & Hua, 2011). In this sense, future studies can extend the findings reported here by enrolling a more representative sample, especially middle-age and elderly participants with lower levels of formal education.

Overall, the results obtained here offer the first evidence of validity and reliability for the European Portuguese translation of the PRMQ. This is an important step since there are few available measures in Portuguese for the assessment of episodic memory complaints.

## Additional File

The additional file for this article can be found as follows:

- pb-58-1-387-s1.pdf
  **Supplementary Figure 1** Supporting Table S1 and Supporting Figures S1–S3. DOI: https://doi.org/10.5334/pb.387.s1
