# Supplementary material for: Subjective Memory Complaints in Portuguese Young Adults: Contributions from the Adaptation of the Prospective and Retrospective Memory Questionnaire
Source: Psychol Belg. 2018 Apr 27;58(1):91–104. doi: 10.5334/pb.387 (PMC6194516; doi:10.5334/pb.387)
Supplement: Supplementary file 1 [file pb-58-1-387-s1.pdf]

Supporting Information

Supporting Table S1

Brief Systematization of the Reliability Properties Reported in Other Adaptation Studies of the PRMQ

Study	Participants				Reliability properties
	N	Sex	Age *M* ± *SD*	Formal education *M* ± *SD*	
Smith et al., 2000	155 AD	106f	73.95 ± 8.77	12.14 ± 3.60	IC = 0.84
	155 caregivers	96f	56.85 ± 13.20	15.51 ± 3.55	
	232 OA	170f	72.74 ± 8.30	12.97 ± 3.39	
	164 YA	102f	44.19 ± 11.40	14.04 ± 3.70	
Crawford et al., 2003	551	344f	63.62 ± 15.59	13.22 ± 3.38	IC: Total = 0.89; PM = 0.84; RM = 0.80
Crawford et al., 2006	570	302f	Raters: 40.70 ± 16.68<br>Ratees: 42.60 ± 17.02	n/a	IC (proxy rating-version): Total = 0.92; PM = 0.87; RM = 0.83
Benites and Gomes, 2007	642	405f	26.62 ± 13.89	13.82 ± 3.02	IC (PRMQ-10): Total = 0.80;<br>Subsample of OA: Total = 0.87; PM = 0.78; RM = 0.76
Rönnlund et al., 2008	540	273m	n/a (age: 35-90)	n/a	IC: Total = 0.89; PM = 0.86; RM = 0.78
Gondo et al., 2010	549 Y	253m	19.60 ± 1.30	n/a	IC: PM = 0.85; RM = 0.84;<br>Y PM = 0.79; Y RM = 0.76;
	1291 YO	704m	65.00 ± 3.10	13.60 ± 2.50	YO PM = 0.87; YO = 0.85;
	860 OA	582m	74.00 ± 3.70	13.10 ± 2.70	OA PM = 0.87; OA RM = 0.87
Piauilino et al., 2010	664	341f	n/a (age: 20-79)	n/a	IC: Total = 0.89; PM = 0.85; RM = 0.77

González-Ramírez & Mendoza-González, 2011	520	302m	39.32 ± 12.70	n/a	IC: Total = 0.89; PM = 0.84; RM = 0.76; Split-half reliability: Total = 0.87; TRR: Total = 0.81; PM = 0.78; RM = 0.80
Hsu & Hua, 2011	48 YA	n/a	n/a (≤35)	n/a	IC (11-item version): Total = 0.90; PM = 0.86; RM = 0.82
	221 OA	n/a	n/a (≥36) Overall = 61.56 ± 19.12	n/a Overall = 11.08 ± 5.11	
van der Werf & Vos, 2011	425	295f	40.02 ± 14.78	n/a	IC: Non-clinical/Clinical Total = 0.92/0.93; Non-clinical/Clinical Total PM = 0.82/0.83; Non-clinical/Clinical Total RM = 0.90/0.90
	217 (clinical population)	135m	54.94 ± 13.87	n/a	
Hsu et al., 2014	140 (neurological outpatients, informants, 90 with AD)	n/a	No dementia: 65.14 ± 8.65	7.86 ± 4.20	IC: Total = 0.97; PM = 0.94; RM = 0.94
			Very mild dementia: 72.02 ± 8.79	5.39 ± 3.43	
			Mild dementia: 79.25 ± 7.33	5.40 ± 4.30	
Talbot & Kerns, 2014	36 children (ADHD)	27m	10.86 ± 1.52	n/a	IC (PRMQ for children): Total = 0.93; PM = 0.91; RM = 0.81
	33	16f	10.89 ± 1.76		
Thompson et al., 2015	53	31f	77.60 ± 4.70	11.70 ± 3.30	IC: Control/Informant PM = 0.86/0.87; Control/Informant RM = 0.79/0.82; MCI/Informant PM = 0.86/0.92; MCI/Informant RM = 0.71/0.81; Dementia/Informant PM = 0.81/0.92; Dementia/Informant RM = 0.77/0.89
	48 (MCI)	26m	78.60 ± 4.90	11.70 ± 3.70	
	37 (dementia)	19m	80.10 ± 5.70	11.40 ± 3.40	
	117 (informants)	n/a	Controls: 63.30 ± 13.00 MCI: 63.20 ± 14.20 Dementia: 60.10 ± 14.20	n/a	

Note. AD = Alzheimer's Disease; ADHD = Attention Deficit Hyperactivity Disorder; IC = Internal Consistency; MCI = Mild Cognitive Impairment; n/a = Not Available; OA = Older Adults; PM = Prospective Memory; PRMQ = Prospective and Retrospective Memory Questionnaire; RM = Retrospective Memory; TRR = Test-rest Reliability; Y = Young; YA = Younger Adults; YO = Young Old.

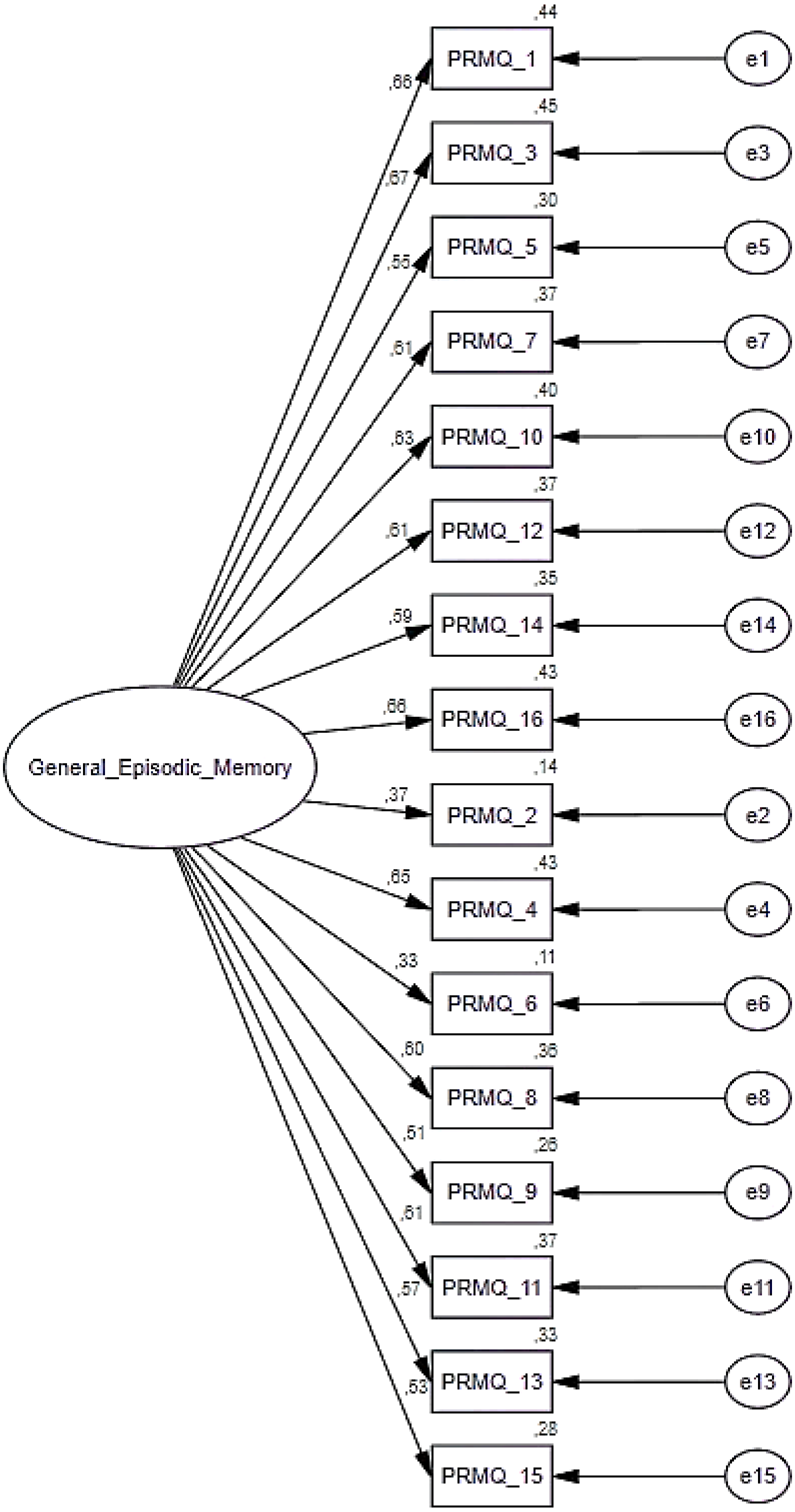

Supporting Figure S1. Graphic representation of the Model 1 in which a general episodic memory factor accounted for the covariance matrix of all 16 items.

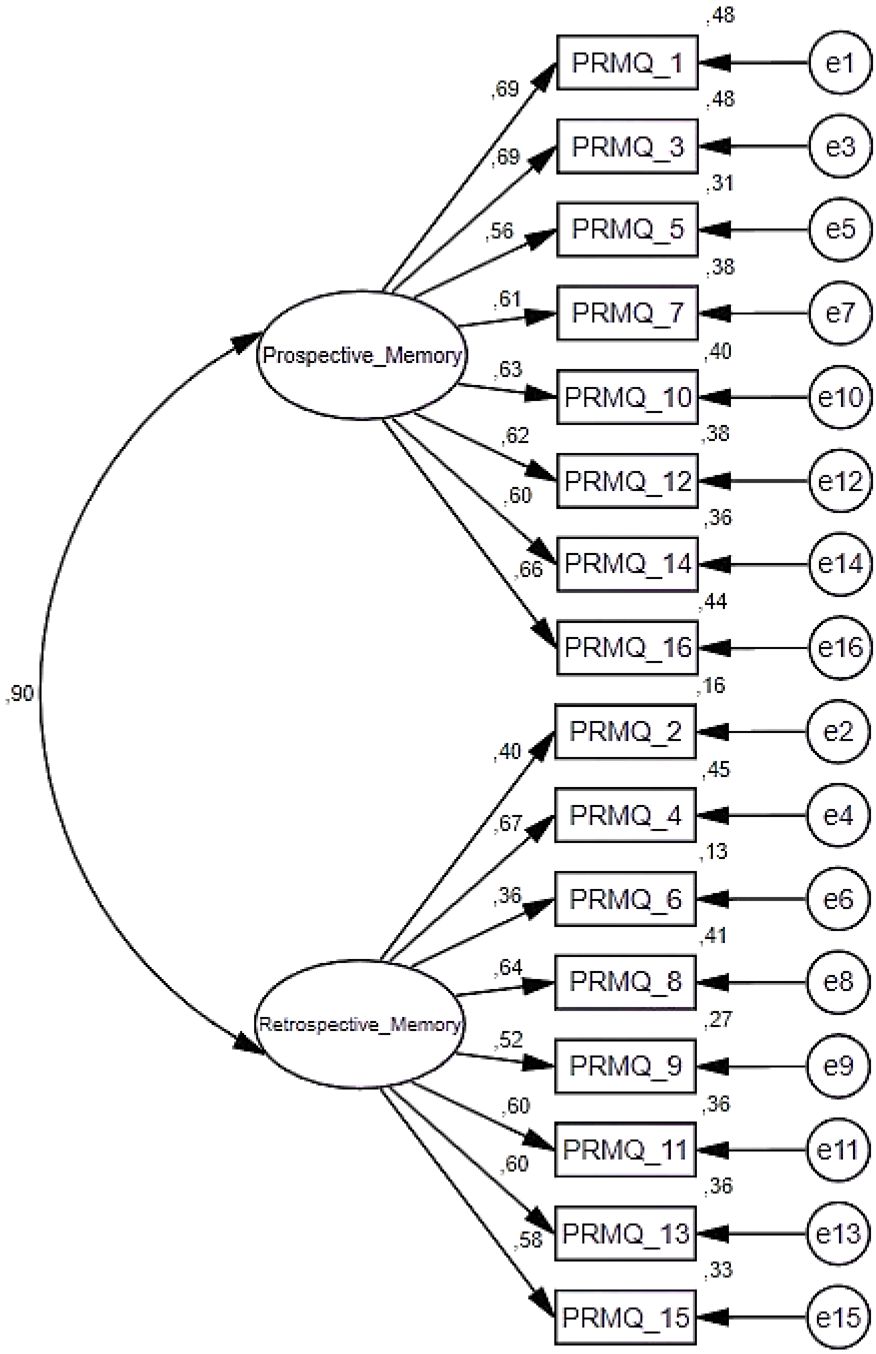

Supporting Figure S2. Graphic representation of the Model 2 with two correlated factors, a PM factor and RM factor.

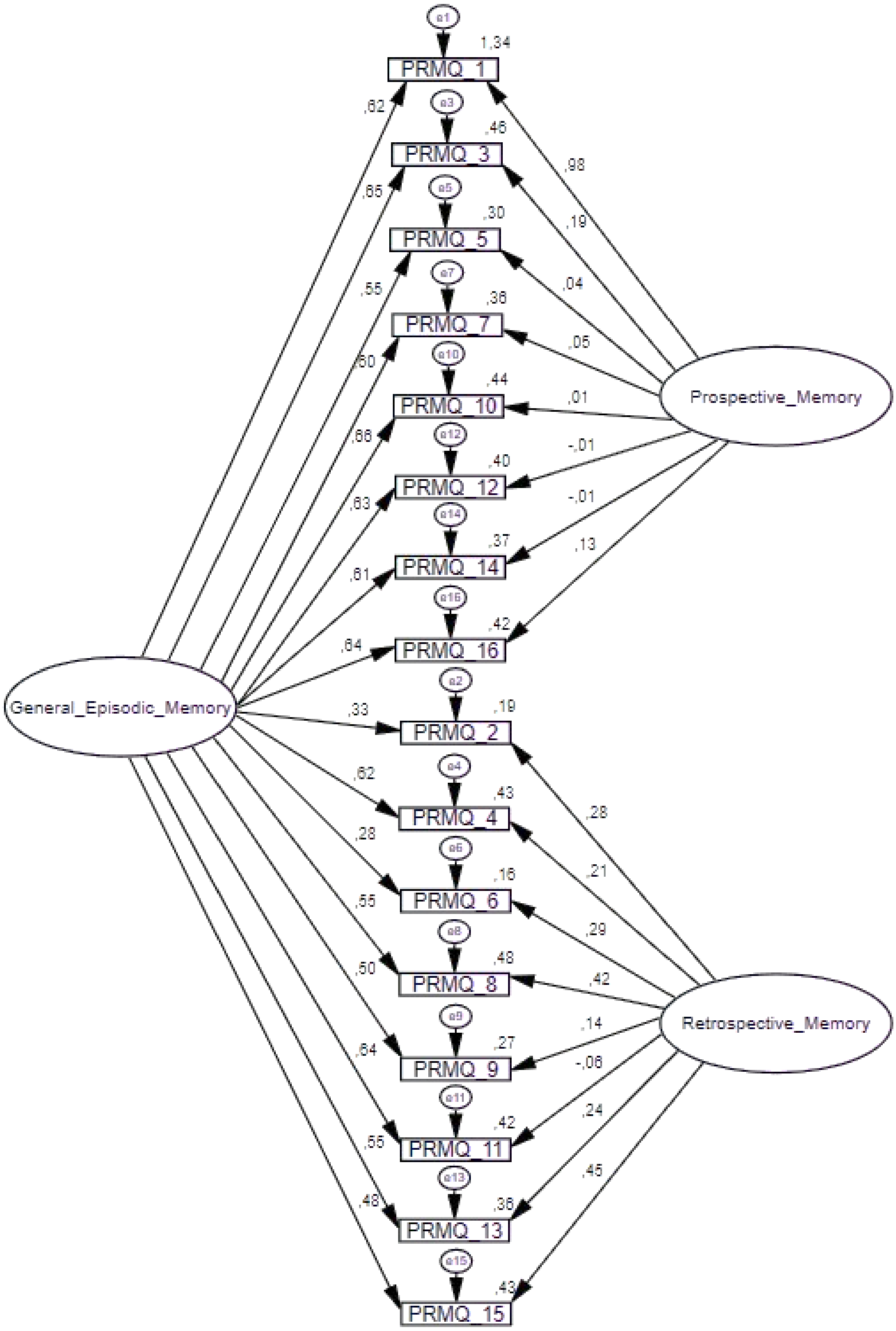

Supporting Figure S3. Graphic representation of the Model 3 with a general episodic memory factor, a PM factor and a RM factor.
